# Coronavirus Disease (COVID-19): The Value of Chest Radiography for Patients Greater Than Age 50 Years at an Earlier Timepoint of Symptoms Compared With Younger Patients

**DOI:** 10.31486/toj.20.0102

**Published:** 2021

**Authors:** Mae Igi, Molly Lieux, Joe Park, Catherine Batte, Bradley Spieler

**Affiliations:** ^1^ Department of Diagnostic Radiology, Louisiana State University Health Sciences Center, New Orleans, LA; ^2^ Louisiana State University Health Sciences Center, School of Medicine, New Orleans, LA; ^3^ Department of Physics and Astronomy, Louisiana State University, Baton Rouge, LA

**Keywords:** *Chest radiography*, *COVID-19*, *diagnostic imaging*, *pneumonia*, *x-rays*

## Abstract

**Background:** A relative paucity of data exists regarding chest radiography (CXR) in diagnosis of coronavirus disease (COVID-19) compared to computed tomography. We address the use of a strict pattern of CXR findings for COVID-19 diagnosis, specifically during early onset of symptoms with respect to patient age.

**Methods:** We performed a retrospective study of patients under investigation for COVID-19 who presented to the emergency department during the COVID-19 outbreak of 2020 and had CXR within 1 week of symptoms. Only reverse transcription polymerase chain reaction (RT-PCR)–positive patients were included. Two board-certified radiologists, blinded to RT-PCR results, assessed 60 CXRs in consensus and assigned 1 of 3 patterns: characteristic, atypical, or negative. Atypical patterns were subdivided into more suspicious or less suspicious for COVID-19.

**Results:** Sixty patients were included: 30 patients aged 52 to 88 years and 30 patients aged 19 to 48 years. Ninety-three percent of the older group demonstrated an abnormal CXR and were more likely to have characteristic and atypical–more suspicious findings in the first week after symptom onset than the younger group. The relationship between age and CXR findings was statistically significant (χ^2^ [2, n=60]=15.70; *P*=0.00039). The relationship between negative and characteristic COVID-19 CXR findings between the 2 age cohorts was statistically significant with Fisher exact test resulting in a *P* value of 0.001.

**Conclusion:** COVID-19 positive patients >50 years show earlier, characteristic patterns of statistically significant CXR changes than younger patients, suggesting that CXR is useful in the early diagnosis of infection. CXR can be useful in early diagnosis of COVID-19 in patients older than 50 years.

## INTRODUCTION

The coronavirus disease (COVID-19) pandemic of 2020, caused by severe acute respiratory syndrome coro-navirus 2 (SARS­CoV­2, initially referred to as 2019­nCoV), has resulted in challenges to essentially all sectors of global society^1^ and particularly to the health care sector.^2^ The radiology community responded with a plethora of publications during the relatively short interval of rapid case increases at the beginning of 2020. Experts across the globe, including the Radiological Society of North America (RSNA) and the American College of Radiology,^3,4^ provided recommendations on the utility of imaging in diagnosis and management of COVID-19.

Most of these recommendations, however, focus on computed tomography (CT) manifestations and not on chest radiographs (CXRs), with the prevailing theme that CT should be used sparingly, with a predilection for patients in the inpatient setting and not as a screening tool.^3-5^ Further, much attention was given to the temporal progression of disease as seen on CT,^6-14^ specifically the increase in multiplicity and density of airspace opacities, ultimately coalescing into diffuse pulmonary opacification and directly correlating with worsening clinical symptoms.^7^ Disease progression from a CXR perspective was addressed relatively less.^15-18^ Some authors underscore their observations with respect to patient age, as incidence and severity of clinical outcomes appear to be greater in older individuals infected with SARS-CoV-2.^19,20^ In fact, 2020 publications from Li et al and Chen et al suggest that the majority of patients they studied with COVID-19 were older than 50 years and that disease progression appears to become more rapid with advances in age.^7,21^

This greater focus on CT is not surprising given the lower sensitivity of CXR vs CT for pulmonary disease in general. However, some centers have used CXR as an adjunct to clinical diagnosis in the early stages of COVID-19, given challenges such as prolonged turnaround time and test variability for reverse transcription polymerase chain reaction (RT-PCR) tests and infection issues related to CT scanning.^22-25^ Wong et al reported the sensitivity of CXR in detecting COVID-19 to be 69% compared to 91% for RT-PCR,^16^ but in the Wong et al study, radiologic evaluation was not geared toward the typical patterns of reported imaging manifestations of COVID-19 described in recent (2020) literature^17,18^ and in consensus statements from the RSNA.^3^ Instead, the study focused on the assessment of pulmonary edema and used a radiographic assessment of lung edema score.^26^

In this article, we address the utility of using a strict pattern of findings on CXR for the diagnosis of COVID-19 during early onset of symptoms in patients older than and younger than 50 years of age.

## METHODS

Our institutional review board approved this retrospective study with waiver of Health Insurance Portability and Accountability Act authorization in accordance with federal regulations at 45 CFR §164.512(i)(2)(ii). Using the keyword “COVID,” we queried the picture archiving and communication system for patient CXRs performed from January 1, 2020 to April 8, 2020. We then searched the medical record numbers associated with the CXRs in the electronic medical record system at University Medical Center in New Orleans, Louisiana, for patients who had undergone COVID-19 RT-PCR assay. Only patients who tested positive by RT-PCR during the same admission were included in the study. Patients with CXRs performed after 7 days of symptom onset were excluded so that we could narrow our assessment of CXR findings to the earliest stages of disease. Symptoms reported by patients at the time of presentation included fever, chills, night sweats, cough, shortness of breath, malaise, loss of appetite, and gastrointestinal complaints.

The query returned 275 CXRs, and 197 patients were confirmed to be RT-PCR positive. From the 197 patients who were RT-PCR–positive, 30 CXRs from patients >50 years and 30 CXRs from patients ≤50 years were chosen. All CXRs were confirmed to have been obtained within 7 days from onset of symptoms. Sex, age, and date of symptom onset were collected for the cohort.

### Image Acquisition and Analysis

All CXRs were acquired as digital radiographs following usual institutional protocols using a Philips DigitalDiagnost system (Koninklijke Philips N.V.). The CXRs were acquired in anteroposterior projection with patients in upright (22/60, 37%), semi-upright (29/60, 48%), or supine (9/60, 15%) position, depending on the patients’ dispositions at the time of image acquisition.

### Data Interpretation

Two board-certified radiologists (with more than 5 and 10 years of experience interpreting CXRs following completion of residency) were blinded to RT-PCR results and clinical history and retrospectively reviewed 60 CXRs in 60 patients obtained on average within 3 days of symptom onset. The two reviewers independently assigned one of the following patterns to each CXR: characteristic, atypical, or negative (Table 1). Discrepancies in the independent interpretations were resolved in a consensus session.

**Table 1.** Chest Radiograph Imaging Classifications^16,27-29^

Classification	Description
Characteristic COVID-19 appearance	Presence of commonly reported chest imaging findings associated with COVID-19: bilateral patchy or confluent band-like ground glass opacity or consolidation in a peripheral and mid-to-lower lung zone distribution
Atypical–more suspicious than not for COVID-19	Presence of some but not all of the characteristic findings
Atypical–less suspicious for COVID-19	Findings suggestive of a diagnosis other than COVID-19 infection (eg, pulmonary edema, atelectasis, interstitial changes)
Negative	No abnormal findings

**Figure 1.**
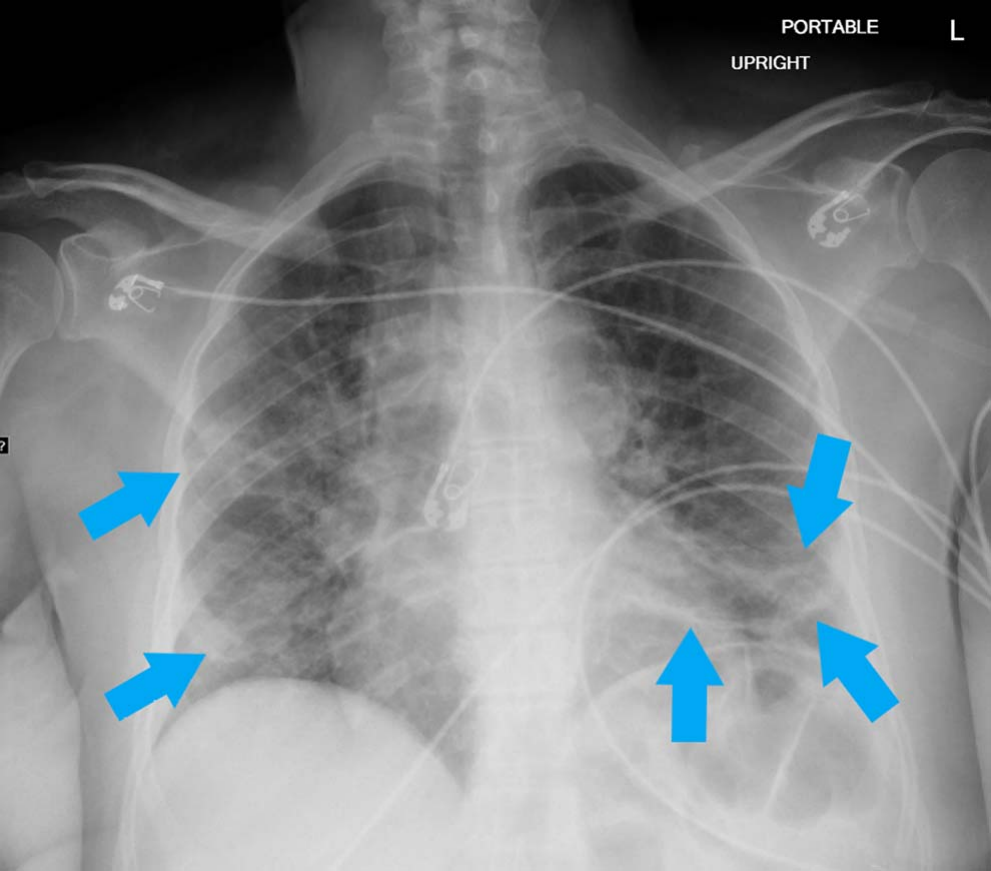
Portable upright anteroposterior radiograph of a 65-year-old female shows characteristic bilateral confluent, bandlike (arrows) consolidative opacity in a peripheral, mid-to-lower lung zone distribution admixed with patchy airspace opacity.

**Figure 2.**
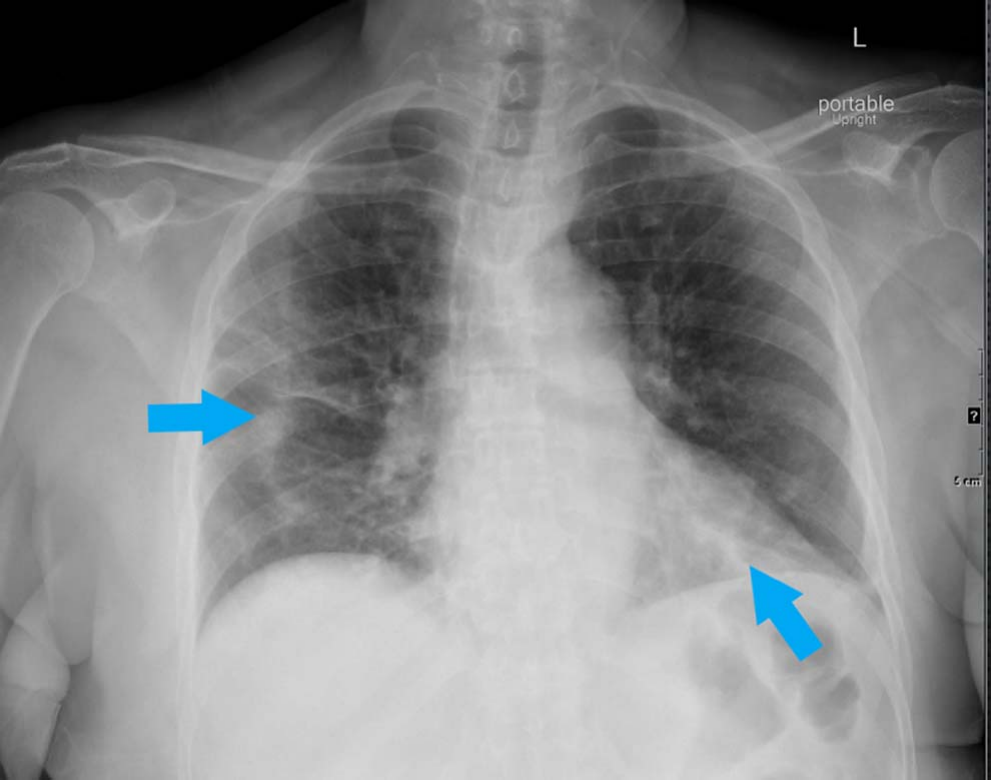
Portable upright anteroposterior radiograph of a 72-year-old female shows characteristic bilateral confluent, bandlike (arrows) consolidative opacity in periphery of the right mid- and left-lower lung zones.

**Figure 3.**
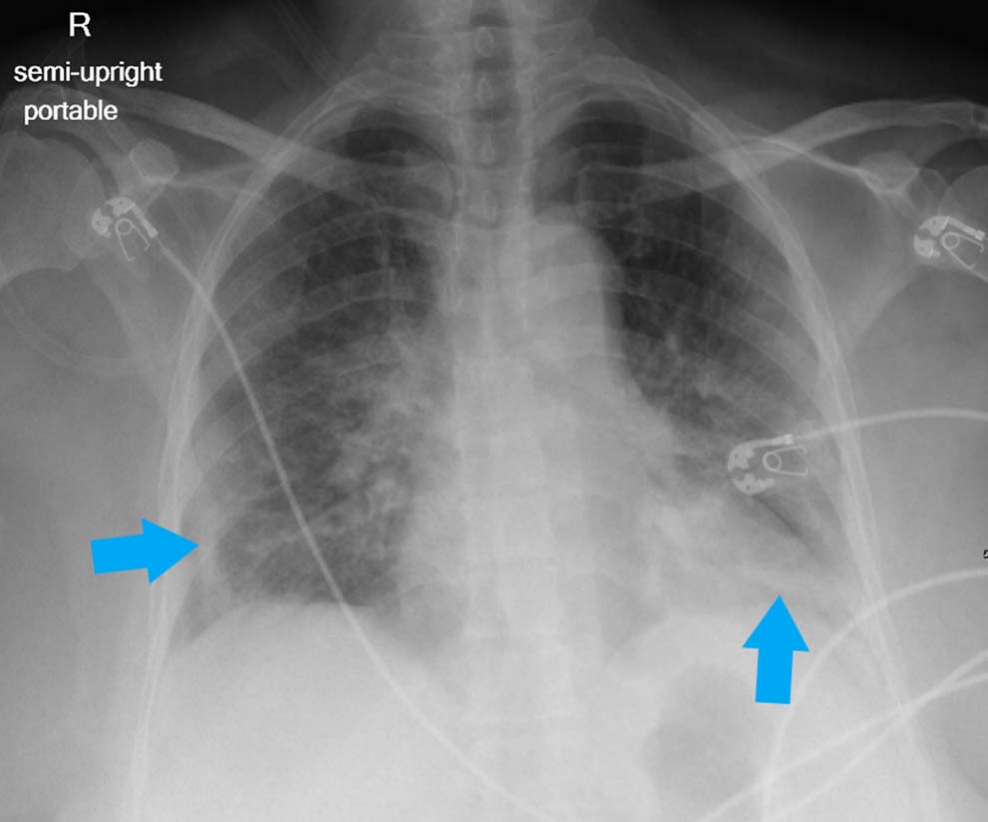
Portable semi-upright anteroposterior radiograph of a 66-year-old female shows characteristic bilateral confluent, bandlike (arrows) consolidative opacity in a peripheral, mid-to-lower lung zone distribution admixed with patchy airspace opacity, greatest at the left lung base.

**Figure 4.**
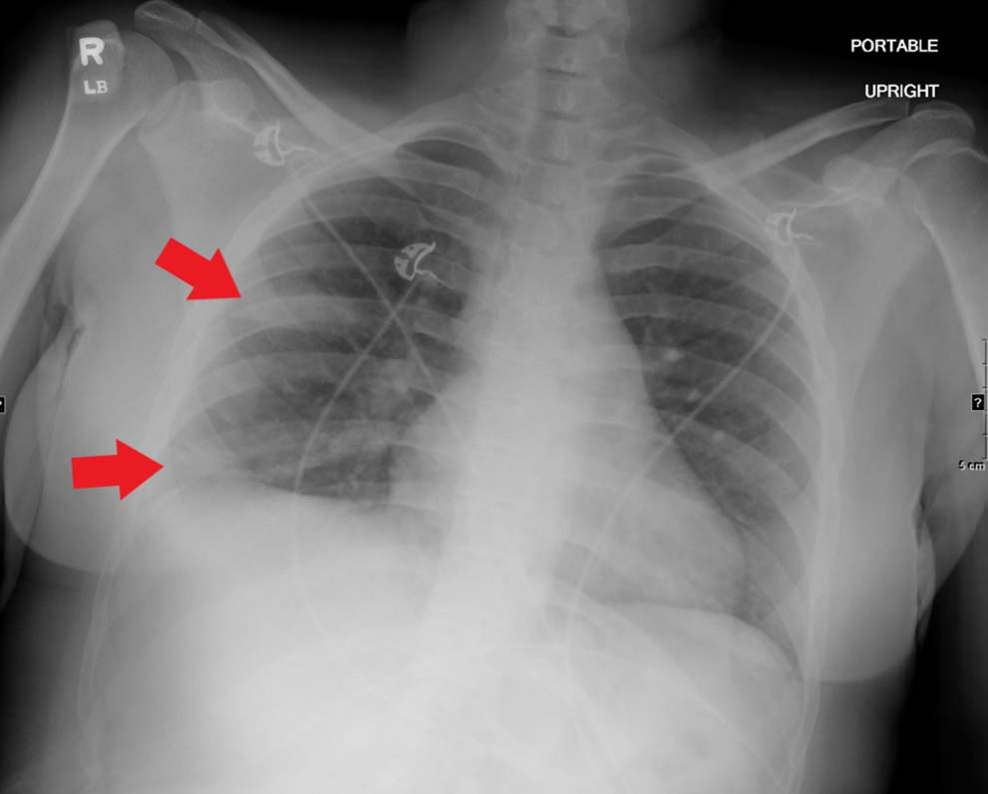
Portable upright anteroposterior radiograph of a 30-year-old female shows unilateral confluent, bandlike (arrows) consolidative opacity in the periphery of the right mid and lower lung zones. This pattern was considered atypical, but more suspicious for coronavirus disease 2019.

**Figure 5.**
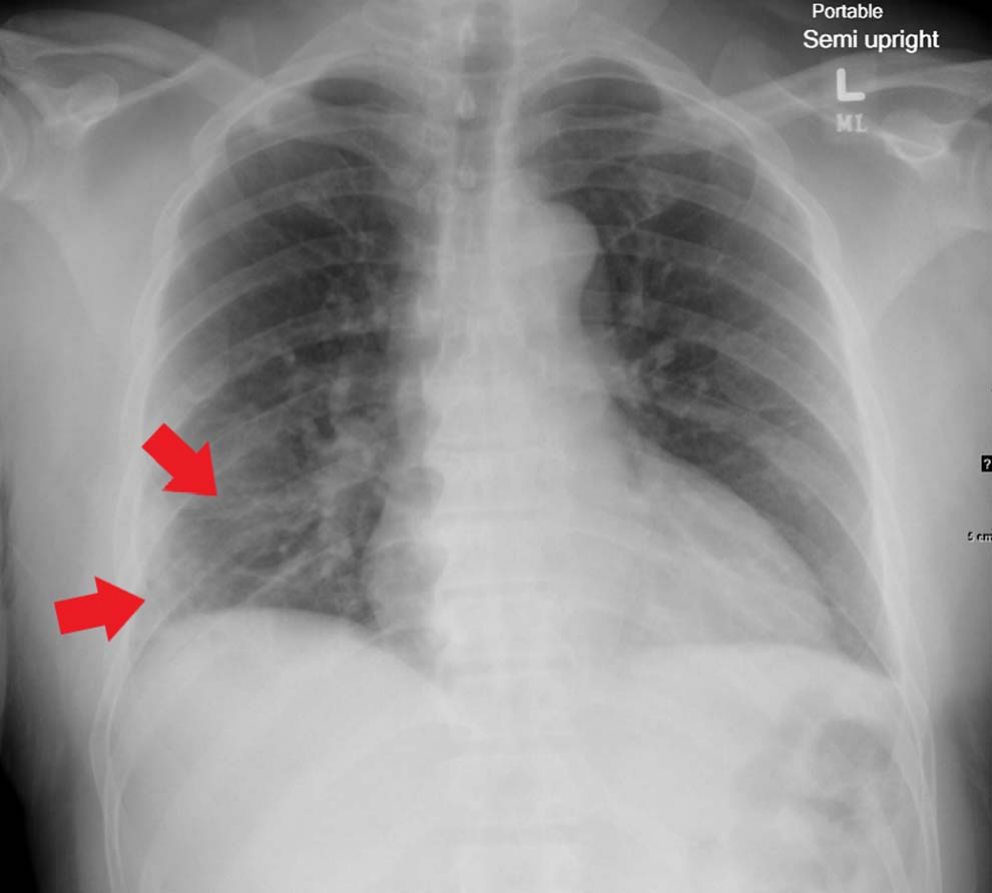
Portable semi-upright anteroposterior radiograph of a 56-year-old male shows unilateral thin bandlike (arrows) consolidative opacity in the periphery of the right mid and lower lung zones admixed with patchy airspace opacity. This pattern was considered atypical, but more suspicious for coronavirus disease 2019.

**Figure 6.**
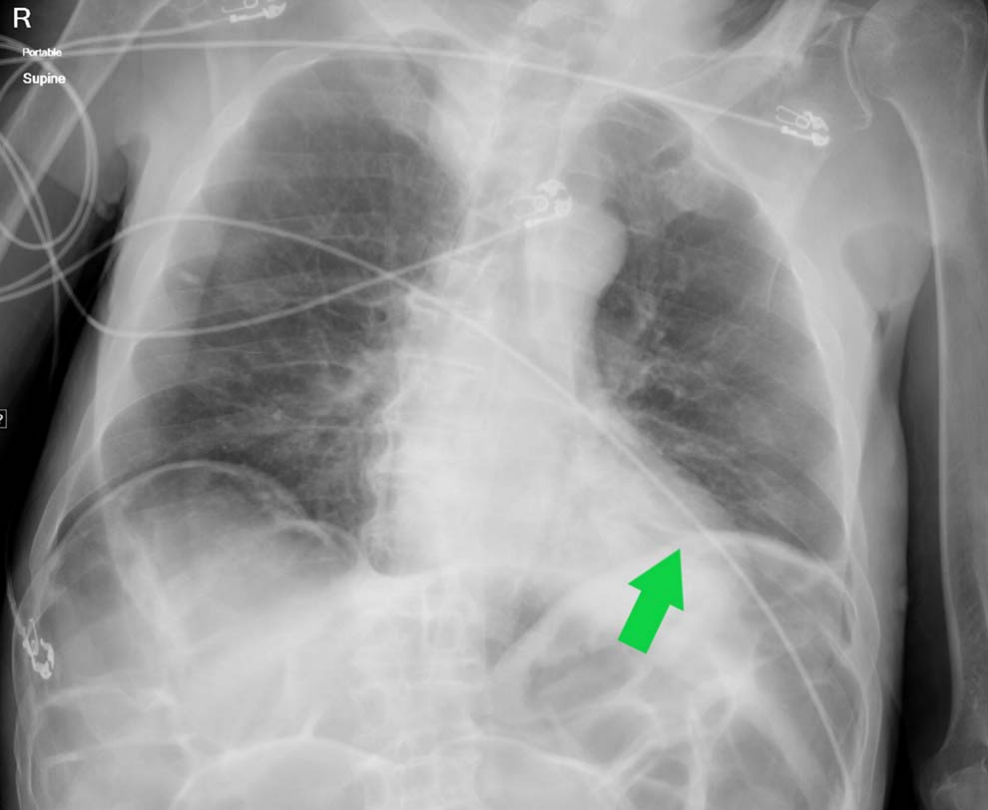
Portable supine anteroposterior radiograph of an 80-year-old male shows unilateral discoid (arrow) opacity along the left hemidiaphragm, typical of subsegmental atelectasis. This pattern was considered atypical and less suspicious for coronavirus disease 2019.

**Figure 7.**
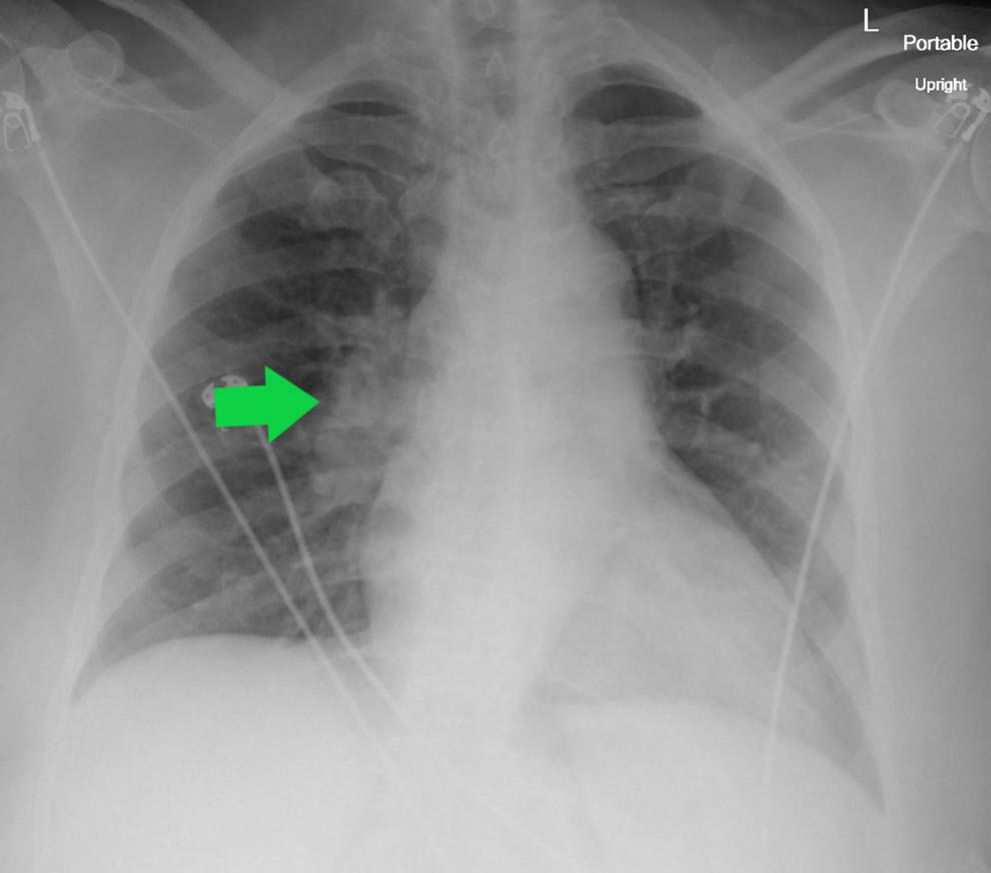
Portable upright anteroposterior radiograph of a 68-year-old female shows prominence of the right hilar shadow (arrow) and otherwise clear lungs. This pattern was considered atypical and less suspicious for COVID-19.

The characteristic COVID-19 pattern was defined in accordance with the most commonly reported chest imaging findings of COVID-19 in recent (2019 and 2020) literature, including the presence of bilateral patchy or confluent, bandlike ground glass opacity or consolidation in a peripheral and mid-to-lower lung zone distribution (Figures 1, 2, and 3).^16,27-29^ If the CXR showed some but not all these abnormalities, it was assigned to the atypical category. CXRs in the atypical category were subdivided into categories based on imaging findings being *more suspicious than not for COVID-19 or less suspicious for COVID-19.* The atypical–more suspicious CXRs had a characteristic pattern seen only in one lung (Figures 4 and 5). The atypical–less suspicious CXRs were defined as abnormal but not necessarily showing a typical finding for COVID-19 pneumonia (Figures 6 and 7). A normal-appearing CXR was categorized as negative.

### Statistical Analysis

Statistical analysis was performed using Excel 2018 (Microsoft Corporation). CXR characterizations—negative, atypical–more suspicious, atypical–less suspicious, and characteristic—were compared to patient age with a threshold of 50 years using a chi-square test of independence and Fisher exact test. Significance level was defined as *P*<0.05.

Interreader agreement was assessed using Cohen kappa (κ) coefficient. The 2 readers had substantial agreement on the evaluation of CXRs for COVID-19 (κ=0.743), agreeing on 50 of the 60 (83%) CXRs.^30^

## RESULTS

Of the 60 patients included in the study, 30 (10 males, 20 females) were in the >50 years group and 30 (14 males, 16 females) were in the ≤50 years group (Table 2). The range of ages in the >50 years group was 52 to 88 years (mean, 65 years). The range in the younger group was 19 to 48 years (mean, 37 years). All 60 patients had a positive COVID-19 RT-PCR assay. Both the older and younger cohorts received CXRs an average of 3 days after the reported onset of symptoms, with a range of 0 to 5 days in the older group and a range of 1 to 7 days in the younger group.

**Table 2.** Imaging Results by Age Group and by Sex by Age Group

	Chest Radiograph Findings^a^			
		Characteristic	Atypical–More	Atypical–Less
Variable	Negative	COVID-19 Appearance	Suspicious	Suspicious
Age, years				
>50 (n=30)	2 (3)	11 (18)	13 (22)	4 (7)
≤50 (n=30)	14 (23)	4 (7)	5 (8)	7 (12)
Sex by age group, years				
Male >50 (n=10)	1 (2)	3 (5)	5 (8)	1 (2)
Female >50 (n=20)	1 (2)	8 (13)	8 (13)	3 (5)
Male ≤50 (n=14)	8 (13)	3 (5)	0 (0)	3 (5)
Female ≤50 (n=16)	6 (10)	1 (2)	5 (8)	4 (7)

Note: All percentages are calculated based on the total number of patients included in the study (n=60).

^a^Classifications of chest radiograph findings are defined in Table 1.

### Imaging Findings

**Figure 8.**
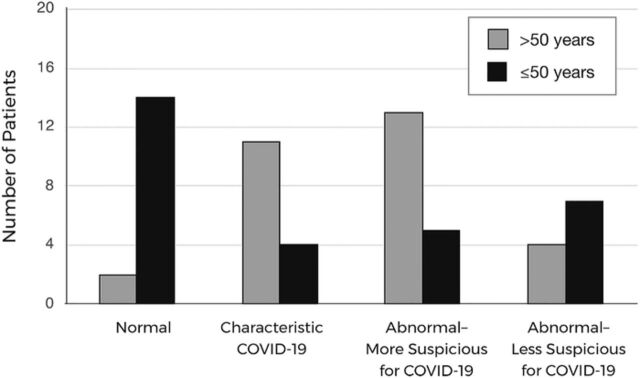
Chest radiograph findings for patients in the 2 age cohorts.

Table 2 shows the imaging findings by classification and by group. Overall, 73% (44/60) of the patients included in this study had an abnormal CXR (Table 3). In the >50 years group, 93% (28/30) had an abnormal CXR that was classified either as characteristic (classic COVID-19 findings) or as atypical. Seven percent (2/30) of patients in the >50 years group had a negative CXR within the first week of presentation from symptom onset. In the same period from symptom onset, 53% (16/30) of patients in the younger group had an abnormal CXR, and the other 47% (14/30) had a normal CXR (Table 3 and Figure 8).

**Table 3.** Overall Imaging Results by Age Group

	Chest Radiograph Findings^a^	
Age Group, years	Normal	Abnormal
>50 (n=30)	2 (7)	28 (93)
≤50 (n=30)	14 (47)	16 (53)

Note: Percentages are calculated by row (n=30).

^a^All negative radiographs are classified as normal. The abnormal category includes radiographs classified as characteristic, atypical–more suspicious, and atypical–less suspicious.

The relationship between age and CXR findings was statistically significant according to chi-square test ([2, n=60]=15.70; *P*=0.00039). In addition, a Fisher exact test comparing negative and characteristic COVID-19 CXR findings to patient age determined that the relationship was statistically significant (*P*=0.001). Patients >50 years were more likely to have characteristic and abnormal suspicious CXR findings during the first week after symptom onset.

## DISCUSSION

Our finding of 73% CXR abnormalities within the first week of symptom onset in the combined cohort closely approximates other reported measures of sensitivity, such as Wong et al at 69%^16^ and Hosseiny et al in which 75% of patients had a similar distribution of findings in early imaging.^31^ When patient age is considered, we demonstrated a 93% sensitivity in patients >50 years and 53% in patients ≤50 years in the first 7 days after symptom onset.

Older age has been associated with poorer outcomes in COVID-19.^31,32^ In the United States, 80% of deaths have occurred in patients ≥65 years, and patients ≥85 years have an overwhelming percentage of severe outcomes, mirroring the reported initial experience in China.^33^

Many associated factors have been addressed in the literature, such as angiotensin-converting enzyme 2 (ACE2) receptor, identified as a target of SARS-CoV-2.^34^ One suggestion is that different levels of ACE2 in younger and older individuals, in particular with respect to decreasing levels within the aging tissues of the lungs and heart, may have an effect on the severity of COVID-19–related disease.^35^ Liu et al reported high levels of angiotensin in correlation with increased viral load of SARS-CoV-2.^36^ In addition, noncommunicable illnesses associated with advancing age, such as hypertension and heart disease, in combination with their associated therapies are generally thought to hinder immune response.^37^ Other age-related links in infectious disease have also been noted. For example, Rivers et al reported that the major risk factors for progression of disease in Middle East respiratory syndrome (MERS-CoV) were underlying comorbidities, such as cardiac disease and diabetes, and age, and the reported mean age in patients considered most severely ill was 57 years.^38^

While a new strain of coronaviridae, SARS­CoV­2, has been identified as the causative pathogen for the COVID-19 pandemic, the outbreak of severe pulmonary disease caused by a strain of this viral family is not unique. MERS-CoV and severe acute respiratory syndrome (SARS-CoV) are the most salient examples. While no outbreaks of SARS-CoV have been reported since April 2004, MERS-CoV cases have been reported as recently as January 2020.^39^ Both syndromes have imaging features similar to COVID-19,^31^ which is important because symptoms associated with COVID-19 have been reported to be milder than those of MERS-CoV,^40^ allowing for a potentially greater role for imaging in disease management, particularly in patients at greater risk for disease severity—the older population.

Much is still unknown regarding patients’ future immunity to COVID-19 after convalescence. The immune system of older adults undergoes numerous age-related changes, collectively termed immune senescence, that leave older adults particularly vulnerable to new, emerging infectious diseases, such as possible reinfection by COVID-19 or infection with new variants.^33^

Limitations of our study include the small sample size at a single institution, lack of follow-up serial CXR or subsequent CT scans that may have been performed, and inclusion of only RT-PCR–positive patients. Selection bias was also present because all patients were chosen from a subset that received imaging, potentially because of a more severe course of illness. Additionally, descriptions of the temporal progression of disease used in this study have been defined almost entirely by recent publications on CT findings. With the rapid increase in number of abnormalities and distribution peaking 6 to 11 days after onset, identifying how patients with a worsening course presented on early CXR will be important.^14^ Despite global vaccination efforts, there is still concern that the disease could produce enough severe illness to further strain some health care infrastructures, especially with new variants circulating in the community. As a result, health care providers need to be able to predict which patients will have a more complex course of illness.^41^ Early CXR correlation with patient course of illness and outcomes could be critical in anticipating what resources will be needed in the coordination of care for COVID-19 patients.

## CONCLUSION

We conclude that CXR in patients >50 years within 1 week of symptom onset in COVID-19 offers potential benefits, whereas a negative CXR in patients ≤50 years has limited sensitivity. Imaging obtained early in presentation of disease, especially for patients older than 50 years, may help to inform an estimate of resource use.

## Notes

*This article meets the Accreditation Council for Graduate Medical Education and the American Board of Medical Specialties Maintenance of Certification competencies for Patient Care, Medical Knowledge, and Practice-Based Learning and Improvement.*
